# Supplementary material for: Dynalign II: common secondary structure prediction for RNA homologs with domain insertions
Source: Nucleic Acids Res. 2014 Nov 21;42(22):13939–48. doi: 10.1093/nar/gku1172 (PMC4267632; doi:10.1093/nar/gku1172)
Supplement: SUPPLEMENTARY DATA [file supp_gku1172_nar-02021-z-2014-File012.zip › manual/GUI/html/ProbKnot.html]

RNAstructure GUI Help -- ProbKnot


|  |  |  |
| --- | --- | --- |
|  | RNAstructure GUI Help ProbKnot | - Contents - Index |
| ProbKnot uses base pair probabilities determined using a prior partition function calculation to predict structures that can contain pseudoknots.  **Predicting Pseudoknots in a Structure**   1. Click the button labeled "Partition Function Save File." A dialog box will open to get the name of a partition function save file (which may or may not contain pseudoknots). 2. A default name is provided for the output ct structure file containing the predicted structure. This can be changed by clicking the "Output CT File" button. 3. Change the number of iterations the calculation undergoes or the minimum allowed helix length for pseudoknots, if desired. The default parameters work best for most situations. 4. Press the "Start" button. 5. When the calculation is complete, a dialog box will open to ask if you would like the predicted structure draw on the screen. | | |
| Visit The Mathews Lab RNAstructure Page for updates and latest information. | | |
